# Robust prognostic value of a knowledge-based proliferation signature across large patient microarray studies spanning different cancer types

**DOI:** 10.1038/sj.bjc.6604746

**Published:** 2008-11-04

**Authors:** M H W Starmans, B Krishnapuram, H Steck, H Horlings, D S A Nuyten, M J van de Vijver, R Seigneuric, F M Buffa, A L Harris, B G Wouters, P Lambin

**Affiliations:** 1 Maastricht Radiation Oncology (Maastro), GROW Research Institute, University of Maastricht, Uns 50/23, PO box 616, Maastricht 6200MD, The Netherlands; 2 Department of CAD and Knowledge Solutions, Siemens Medical Solutions PA, 51 Valley Stream Pkwy E51, Malvern, PA 19355, USA; 3 Department of Experimental Therapy, The Netherlands Cancer Institute, Plesmanlaan 121, Amsterdam 1066CX, The Netherlands; 4 Department of Diagnostic Oncology, The Netherlands Cancer Institute, Plesmanlaan 121, Amsterdam 1066CX, The Netherlands; 5 Department of Radiation Oncology, The Netherlands Cancer Institute, Plesmanlaan 121, Amsterdam 1066CX, The Netherlands; 6 Department of Molecular Oncology Laboratories, Weatherhall Institute of Molecular Medicine, Cancer Research UK, University of Oxford, Old Road Headington, Oxford OX3 9DS, UK; 7 Ontario Cancer Institute/Princess Margaret Hospital, 610 University Ave, Toronto, Canada ON M5G 2M9

**Keywords:** gene signature, gene expression profiling, microarray, proliferation, prognostic marker

## Abstract

Tumour proliferation is one of the main biological phenotypes limiting cure in oncology. Extensive research is being performed to unravel the key players in this process. To exploit the potential of published gene expression data, creation of a signature for proliferation can provide valuable information on tumour status, prognosis and prediction. This will help individualising treatment and should result in better tumour control, and more rapid and cost-effective research and development. From *in vitro* published microarray studies, two proliferation signatures were compiled. The prognostic value of these signatures was tested in five large clinical microarray data sets. More than 1000 patients with breast, renal or lung cancer were included. One of the signatures (110 genes) had significant prognostic value in all data sets. Stratifying patients in groups resulted in a clear difference in survival (*P*-values <0.05). Multivariate Cox-regression analyses showed that this signature added substantial value to the clinical factors used for prognosis. Further patient stratification was compared to patient stratification with several well-known published signatures. Contingency tables and Cramer's V statistics indicated that these primarily identify the same patients as the proliferation signature does. The proliferation signature is a strong prognostic factor, with the potential to be converted into a predictive test. Furthermore, evidence is provided that supports the idea that many published signatures track the same biological processes and that proliferation is one of them.

The abilities to predict outcome and to identify key players in biological mechanisms that lead to poor outcome are two important objectives in cancer research. Recently, efforts to exploit gene expression profiling have been made to identify gene sets, or so-called gene signatures, that can improve diagnosis and risk stratification (Bild *et al*, 2006). A drawback of most of the studies performed is that supervised analysis methods are utilised to acquire such signatures. In this approach, patient microarray and clinical data are used to find gene sets that correlate with tumour type or survival. This often results in gene sets with a very high prognostic value in the studied data set. However, comparative testing of these signatures in other patient data sets has been limited, and the overlap in selected genes of different comparable studies is small (Chen *et al*, 2007). If such a signature can be applied to other data sets, it may well be restricted to a certain patient population and cancer type. In addition, the gene sets obtained with this method are often difficult to interpret with respect to the underlying biological mechanism (Dai *et al*, 2005; Quackenbush, 2006). Furthermore, Dupuy and Simon (2007) showed in a recent review that many of these studies show flaws in methodology.

An alternative approach to identify prognostic signatures is based on defining gene sets involved in a biological process or specific environmental condition that is suspected of influencing treatment response or patient outcome. In this approach, *in vitro* gene expression profiling is used to identify gene sets that play an important role in a specific biological process. The identified gene set is then applied to gene expression data from patients to evaluate its prognostic value. This approach has a more broad application because the gene sets can be applied in almost every patient group. Furthermore, it can be used not only to investigate whether a certain process is important in a distinct cancer type or patient group but also potentially to select patients in those groups that would be expected to benefit from therapies directed to the biological process of interest (Bild *et al*, 2006). Examples of gene sets attained with this approach are the wound (Chang *et al*, 2004), hypoxia (Chi *et al*, 2006; Sung *et al*, 2007; Winter *et al*, 2007) and ‘invasiveness’ (IGS) (Liu *et al*, 2007) signatures. These studies show that the deduced signatures can be used for risk stratification in very different types of cancers (Chang *et al*, 2004; Chi *et al*, 2006; Liu *et al*, 2007; Winter *et al*, 2007), presumably because of common core pathways that are influencing outcome in these diverse clinical groups. Another potential benefit of this unsupervised approach is that it can potentially identify the functional regulators within a signature that drive the studied process (Adler *et al*, 2006) and thus reveal new targeting candidates. Recently, Fan *et al* (2006) compared the performance of several supervised and unsupervised derived gene sets and found that both types of signatures showed high concordance in prognostic power (van de Vijver *et al*, 2002).

One of the biological processes often implicated in gene expression signatures is cell proliferation. The rate of tumour cell proliferation is a major contributor to treatment response with both chemotherapy and radiotherapy (Bourhis *et al*, 2006) and is reflected in the fact that overall treatment time (e.g., duration of radiotherapy) is an important contributor to outcome (De Ruysscher *et al*, 2006). In a recent review, Whitfield *et al* (2006) showed that proliferation may underlie the prognostic power of many previously identified signatures. He showed that in almost every supervised derived signature a large subset of genes involved in proliferation is included (Perou *et al*, 1999; Dai *et al*, 2005; Sotiriou *et al*, 2006). In some cases, these classifiers have even been designated as ‘proliferation’ signatures, although there derivation was not based on this phenotype. Two of these signatures have recently made it to the clinical setting as a diagnostic tool for patients with breast cancer (van 't Veer *et al*, 2002; Paik *et al*, 2004).

On the basis of these results, we hypothesised that derivation of a specific *in vitro*-derived signature based solely on proliferation may provide valuable information on tumour status, prognosis and outcome prediction across diverse tumour types.

## Materials and methods

### Data sets

Patient microarray and clinical follow-up data were collated to test the clinical value of the signatures. Data sets are publicly available in the microarray databases Gene Expression Omnibus (GEO) and Stanford Microarray Database (SMD) or elsewhere. Accessory clinical and follow-up data were also given or provided by the authors on request. In Table 1A, an overview of the data sets and where they are accessible is provided. Data filtering and pre-processing are explained in the Supplementary Information (Supplementary Materials and Methods). Data sets were imported in Matlab (Matlab 7.1, The Mathworks, Natick, MA, USA). Unless indicated otherwise, analyses were performed in this program.

### Signature score calculation

Expression data of the genes in the signature was extracted from the data set. The following step was used to calculate a signature score for each patient in the data set. This score was defined as the weighted average expression value of the genes in the signature (Equation (1)). A weight of −1 or 1 was assigned to each gene, dependent on the phenotype the gene represented. Weight assignment is described in the results and Supplementary Information (Supplementary Materials and Methods).

The signature score then reflects the status of the studied process in a tumour. When a gene was represented by more than one probe on an array, the expression of the probes was averaged before signature calculation. In Table 1B, the number of signature genes represented in the different data sets is provided for the evaluated signatures. 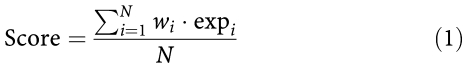 where: score, signature score; *N*, number of genes in the signature; *i*, gene; *w*_*i*_, weight of gene *i*; exp_*i*_, gene expression of gene *i*.

### Statistical analysis

A loop of 1000 clustering repeats with the K-means clustering function in Matlab was applied to split the patients in two groups according to their signature score. Outcome in the two groups was analysed and compared by the Kaplan–Meier method. Differences in outcome were tested for statistical significance by the log-rank test for different common end points. For breast and renal cancer, the common end points are 5- and 10-year survival, and for lung cancer, these are 2- and 5-year survival; all end points were analysed when follow-up was long enough. Results for the log-rank tests are given as the average, standard deviation and the range of the *P*-values, also the percentage of *P*-values from the 1000 clustering runs that were significant was calculated to evaluate the prognostic power of the signature and stability of the clustering.

Multivariate Cox-regression analysis with stepwise backward selection procedure was performed in SPSS (SPSS 12.0.1, SPSS Inc, IL, USA) to show the clinical relevance of the signature.

### AUC model calculation

Matlab was used to integrate all parameters in a model with and without addition of the signature to the clinical parameters. Differences between the models were assessed using receiver–operator curve (ROC) analysis by calculating the AUC. Further details are provided in the Supplementary Information (Supplementary Materials and Methods).

### Random signature testing

A method to test a predefined number of random signatures of a predefined size on all the data sets was developed. To show the strength of a signature, 10 000 random generated gene sets, with sizes equal to the size of the signature of interest, were tested on the data sets. These random gene sets were tested in a similar manner as the other signatures.

### Mitotic index scoring

Mitotic index was assessed (as part of histological grading) in the 295 breast tumours of the van de Vijver *et al* (2002) data set, using a microscope with a field diameter of 0.44 mm with a × 40 objective. The area with the highest mitotic activity was selected and mitotic figures were counted in 10 consecutive fields. Tumours were assigned to the following groups based on the mitotic counts:

- Group 1: 0–5 mitoses in 10 high power fields.
- Group 2: 6–10 mitoses in 10 high power fields.
- Group 3: ⩾11 mitoses in 10 high power fields.

## Results

### Signature derivation

From published microarray studies two different proliferation signatures were compiled. Whitfield *et al* (2002) studied the cell cycle in HeLa cells (cervix cancer cell line). Microarrays were performed on synchronized cell cultures at different time points, and genes that showed a periodic variation were selected. These genes were grouped according to the cell cycle phase in which their expression peaked. We propose that this gene set could be used as a specific proliferation signature.

Another method to derive a proliferation signature with microarrays was used by Chang *et al* (2004). Human fibroblasts were serum starved for 48 h and then stimulated with serum to simulate a wound response. One of the most consistent and important effects in the serum response program is stimulation of proliferation. Abnormal proliferation is also a consistent characteristic of cancer cells, irrespective of a wound response (Chang *et al*, 2004). Chang *et al* (2004) therefore discarded the genes with a periodic behaviour to specifically study the wound response. Here, we propose that the set of genes discarded from the wound signature is a good representation of a proliferation signature. This signature is a subset of the signature derived from Whitfield *et al* (2002); however, we postulate that it is a better representative of proliferation and will be a better prognostic factor, as only this gene set shows a change in expression upon serum stimulation.

The wound (Chang *et al*, 2004) and IGS (Liu *et al*, 2007) signature are two promising published unsupervised derived signatures. Furthermore, the second proliferation signature is derived from the same *in vitro* data as the wound signature. Therefore, these signatures were also analysed.

### Comparison of two proliferation signatures

Signature 1 (Whitfield *et al*, 2002) and signature 2 (Chang *et al*, 2004) consist of respectively 1134 and 199 cloneIDs that map to 815 and 154 unique UnigeneIDs, respectively. The distribution of genes in the different cell cycle phases for the two signatures is distinct (Supplementary Information Table S1), indicating that the signatures are different. Signature 1 shows equal proportions of genes in the defined cell cycle phases. However, in signature 2 more genes are involved in G_2_ and clearly less genes are involved in M/G_1_.

### Outcome prediction with proliferation signatures

The signatures were tested for their clinical relevance on several publicly available microarray data sets (Table 1). Signatures were evaluated using a signature score (Equation (1)), which is defined as a weighted average of the expression of the genes in the signature. To calculate the signature score, weights were defined for each gene. After translating the signatures into UnigeneIDs (build199) and weight assignment, several genes were discarded from analyses, as weight assignment for these genes was ambiguous (details are provided in the Supplementary Materials and Methods). The final signatures consist of respectively 508 and 110 UnigeneIDs for signatures 1 and 2.

**Figure 1:**
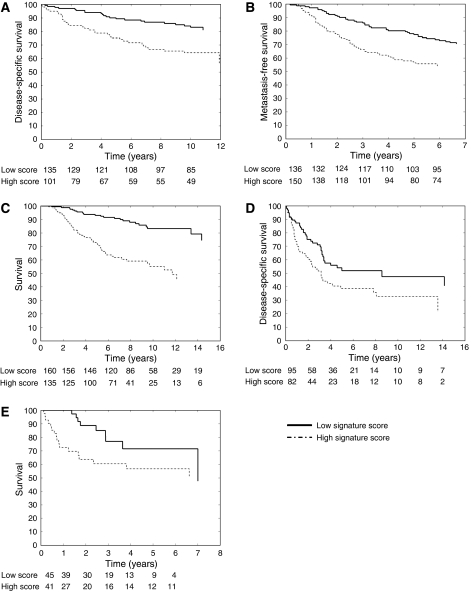
A signature score was calculated for each patient in the different data sets. These scores were used to cluster the patients in two groups, one with low expression and one with high expression of the signature. Kaplan–Meier survival curves for the two groups were compared ((**A**) Miller data set, (**B**) Wang data set, (**C**) van de Vijver data set, (**D**) Zhao data set, (**E**) Beer data set).

In every data set, a signature score (Equation (1)) was calculated for each patient. The patients were separated in two groups by clustering these signature scores, to obtain a natural separation rather than using an arbitrary value such as the median to split the patients. This clustering was repeated 1000 times to assess the stability of the group assignment. Results of the log-rank tests are given in Supplementary Information Table S2, and in Figure 1, the Kaplan–Meier curves for signature 2 are shown. Signature 2 gives clear risk stratification in all data sets, all *P*-values of the 1000 clustering runs <0.05. Results of the log-rank test show not only that signature 2 gives a better risk stratification than signature 1, also the overall robustness of the separation is stronger, indicated by the small standard deviations. Nevertheless, both signatures show very good prognostic value on the three breast cancer data sets. The range and standard deviations of the 1000 clustering runs also show that the results are robust for these data sets and that the splitting patients based on clustering of signature scores is stable.

### Statistical analysis of signature scores

Multivariate Cox-regression analyses were performed to investigate whether the association between the best proliferation signature and outcome was independent of clinical prognostic factors. The variables analysed differed per data set, as different clinical factors are provided (Supplementary Information Table S3). A stepwise backward selection procedure was performed to select the variables that are prognostic factors; the end point is 10 years for breast and renal cancer and 5 years for lung cancer. Follow-up time in the Wang *et al* (2005) data set is not long enough, in that data set 5 years was used. In Table 2, the factors selected with this procedure are given for all the data sets, choosing another end point did not influence the results dramatically (Supplementary Information Table S4). In four out of five data sets, the proliferation signature is included in the model as a prognostic factor of outcome. In three data sets this was highly significant and in the fourth it reached border significance.

### AUC calculations

The area under the receiver–operator curve (AUC) was calculated for each clinical parameter and the best proliferation signature. Results of this analyses show that the proliferation signature has a high AUC compared to the clinical parameters in all data sets (Supplementary Information Table S3).

**Figure 2:**
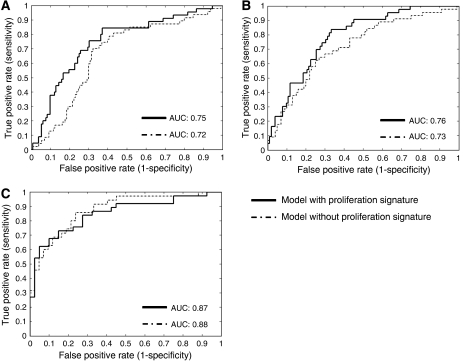
A model of the clinical factors with and without the signature was generated. Receiver–operator curves (ROC) were used to compare the two models in three data sets. ((**A**) Miller data set, (**B**) van de Vijver data set, (**C**) Zhao data set).

To quantify the gain in prognostic power obtained with this signature, a model of the clinical factors with and without the signature was generated and evaluated with the AUC. Part of the data set was used as training set, to generate the model, and the other part as a test set. Only the data sets with more than 1 clinical parameter and more than 150 patients are included. Different sizes of training and test sets were evaluated; the overall performance did not change significantly (data not shown). The results shown in Figure 2 were produced with 150 and 100 samples as training set for the breast cancer and the renal cancer data sets, respectively. In two out of three data sets, the AUC increased significantly when the proliferation signature was added to the model (Figure 2, *P*-values paired *t*-test ≪0.0001).

### Random signature testing

To show the strength of the proliferation signature, 10 000 random generated signatures were tested on all data sets. Of these 10 000, no signature gave a significant result on all data sets.

### Comparison to other signatures

Log-rank tests and Kaplan–Meier survival curves show that the wound and IGS signature give clear risk stratification in four and five data sets, respectively (Supplementary Information Table S2). Furthermore, inclusion of these signatures in multivariate Cox-regression analyses (Supplementary Information Table S4) gives the indication that combining signatures, such as the proliferation and IGS signature in one of the data sets, can strengthen the prognostic power of microarray profiling in a clinical setting. Combining the proliferation and wound signature did not add value in any of the data sets.

To investigate whether different signatures identify the same patients, two-way contingency table analyses (Supplementary Materials and Methods) were performed to compare the patient classification of the proliferation signature to the classification of other signatures. For the gene sets identified in three of the five data sets (Beer *et al*, 2002; van de Vijver *et al*, 2002; Miller *et al*, 2005), group classification was available (details are provided in the Supplementary Materials and Methods); these and the wound and IGS signatures were evaluated. Contingency table analyses and Cramer's V statistics (Supplementary Information Tables S5–S9) show that the proliferation signature has a strong association with all other signatures evaluated, indicating that these signatures identify the same patients.

### Proliferation signature validation

**Figure 3:**
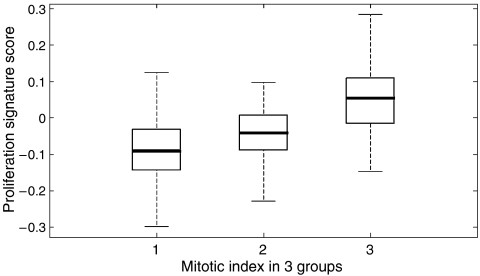
Correlation between the proliferation signature score and the mitotic index in the van de Vijver data set.

The proliferation signature is extracted from *in vitro* data; however, this does not necessarily mean that the signature truly tracks proliferation *in vivo*. To investigate this, mitotic index (MI) was assessed for the van de Vijver *et al* (2002) data set, which was scored in three classes. In Figure 3 a boxplot is shown of the proliferation score *vs* the MI. There is a clear correlation between the average proliferation signature score and the three classes of MI (correlation coefficient: 0.968).

## Discussion

We derived a proliferation signature from *in vitro* microarray studies based only on genes that differ in expression in different parts of the cell cycle (Whitfield *et al*, 2002; Chang *et al*, 2004). Results show that the proliferation signature has a high value in patient risk stratification in five large clinical studies involving more than 1000 patients and three different cancer sites. This contrasts with previous studies that rarely validate signatures in more than one large independent data set.

Our data indicate that the proliferation signature can be combined with other phenotype-based signatures, to further improve patient stratification. The fact that large clusters of proliferation genes are identified in many gene signatures (Perou *et al*, 1999; Rosenwald *et al*, 2003; Dai *et al*, 2005; Dyrskjot *et al*, 2005; Sotiriou *et al*, 2006; Larsen *et al*, 2007; Liu *et al*, 2007) raises the possibility that many of previously reported gene signatures, including the wound signature, may be highly influenced by proliferation. Fan *et al* (2006) has previously suggested that many signatures track a common set of biological phenotypes and therefore have a similar prognostic strength. Whitfield *et al* (2006) has further suggested that one of these processes is proliferation. The performance of the proliferation signature in our study supports this idea. Comparisons of the proliferation signature to five other gene sets showed that these signatures primarily identify the same patients as the proliferation signature.

Some reports refer to their signature as a proliferation signature (Rosenwald *et al*, 2003; Dai *et al*, 2005). However, in these supervised studies not all genes in the signature are related to proliferation and therefore cannot be referred to strictly as general proliferation signatures. For example, Dai *et al* (2005) used a supervised approach to determine a signature associated with metastasis. Many of the identified genes were known to be involved in cell cycle regulation and these authors thus referred to their classifier as a proliferation signature. However, only 17 out of 50 genes in this signature are cell cycle related when assessed in the initial gene list of Whitfield *et al* (2002). The same applies to the study of Rosenwald *et al* (2003), only 28 of the 48 genes that were associated with length of survival are related to proliferation.

The proliferation signature has a high prognostic power, similar to many signatures; however, it is one of the few signatures that may also have a predictive value. It can possibly be used to prescribe a treatment targeting tumour proliferation. Studies indicate that the fast proliferating tumours can benefit from accelerated radiotherapy or chemoradiotherapy (Corvo *et al*, 2000; Gasinska *et al*, 2004). The proliferation signature could possibly be used as basis for a predictive test for patient selection for these treatments.

Previous studies have tried to assess the predictive value of proliferation by means of MI, Ki67 staining and potential doubling time (Tpot) calculation. Overall results of these single-parameter indicators are disappointing (Begg *et al*, 1999). Mitotic index and Ki67 staining are the most promising parameters; however, results for these markers are controversial (Begg *et al*, 1999; Daniels *et al*, 2002; Caly *et al*, 2004; Jalava *et al*, 2006). This can be because of the large chance of misclassification with these single-parameter indicators (Jalava *et al*, 2006; Whitfield *et al*, 2006). Application of multiparameter indicators, such as the proliferation signature, is therefore a more attractive method (Whitfield *et al*, 2006). The proliferation signature shows a clear correlation with MI in one of the tested data sets.

In conclusion, we have shown that the application of phenotype-based signatures such as the proliferation signature can be used in patient risk stratification, in addition to clinical parameters. It has a high prognostic value and unlike other signatures it has the potential to be converted into a predictive test. Furthermore, we provide evidence that supports the idea that many published signatures track the same biological processes and that proliferation is one of them. Whether the proliferation signature can be converted into a predictive test should be evaluated in a large prospective trial in which other measures for proliferation should also be evaluated.

## Figures and Tables

**Table 1:** (A) Overview of the analysed patient microarray data sets. (B) Number of signature genes represented in the microarray data set (number of gene identifiers on the arrays are given between parentheses)

**(A)** **Data set**	**Cancer site**	**No. of patients**	**Source**	
Miller	Breast	251	GEO accession GSE3494: http://www.ncbi.nlm.nih.gov/projects/geo/	
Wang	Breast	286	GEO accession GSE2034: http://www.ncbi.nlm.nih.gov/projects/geo/	
Van de Vijver	Breast	295	http://microarray-pubs.stanford.edu/wound_NKI/	
Zhao	Renal	177	SMD: http://smd.stanford.edu/	
Beer	Lung	86	http://dot.ped.med.umich.edu:2000/ourimage/pub/Lun g/index.html	
				
**(B)** **Data set**	**Signature 1**	**Signature 2**	**Wound signature**	**IGS signature**
Miller	455 (1120)	104 (228)	415 (1030)	176 (516)
Wang	350 (667)	87 (158)	346 (614)	131 (270)
Van de Vijver	192 (242)	51 (59)	171 (195)	67 (87)
Zhao	257 (415)	47 (82)	280 (446)	83 (132)
Beer	192 (224)	45 (51)	171 (195)	63 (76)

**Table 2:** Clinical parameters selected with stepwise backward selection in multivariate Cox-regression analyses including signature 2

	**Hazard ratio (95% CI)**	***P*-value**
*Miller*		
Tumor size	3.3 (1.7–6.6)	0.0006
LNS^a^	2.8 (1.6–5.0)	0.0003
Proliferation^b^	3.4 (1.4–8.2)	0.0052
		
*Wang*		
Proliferation^b^	2.6 (1.5–4.4)	0.0004
		
*Van de Vijver*		
Age	0.95 (0.91–0.99)	0.0096
Tumour size^c^	1.5 (0.93–2.5)	0.0962
Elston grade	2.2 (1.4–3.4)	0.0003
Proliferation^b^	21 (1.8–234)	0.0148
		
*Zhao*		
Performance status	1.3 (1.1–1.6)	0.0069
Grade	1.5 (1.0–2.1)	0.0260
Stage	3.3 (2.5–4.4)	< 0.0001
		
*Beer*		
Age	1.0 (1.0–1.1)	0.0338
Stage	2.3 (1.5–3.5)	0.0002
Proliferation^b^	1.8 (0.9–3.5)	0.0884

a LNS=lymph-node status.

b Proliferation: proliferation signature 2.

c Categories: ⩽2 or >2 cm.

## References

[bib1] Adler AS, Lin M, Horlings H, Nuyten DS, van de Vijver MJ, Chang HY (2006) Genetic regulators of large-scale transcriptional signatures in cancer. Nat Genet 38: 421–430 16518402 10.1038/ng1752 PMC1435790

[bib2] Beer DG, Kardia SL, Huang CC, Giordano TJ, Levin AM, Misek DE, Lin L, Chen G, Gharib TG, Thomas DG, Lizyness ML, Kuick R, Hayasaka S, Taylor JM, Iannettoni MD, Orringer MB, Hanash S (2002) Gene-expression profiles predict survival of patients with lung adenocarcinoma. Nat Med 8: 816–824 12118244 10.1038/nm733

[bib3] Begg AC, Haustermans K, Hart AA, Dische S, Saunders M, Zackrisson B, Gustaffson H, Coucke P, Paschoud N, Hoyer M, Overgaard J, Antognoni P, Richetti A, Bourhis J, Bartelink H, Horiot JC, Corvo R, Giaretti W, Awwad H, Shouman T, Jouffroy T, Maciorowski Z, Dobrowsky W, Struikmans H, Wilson GD (1999) The value of pretreatment cell kinetic parameters as predictors for radiotherapy outcome in head and neck cancer: a multicenter analysis. Radiother Oncol 50: 13–23 10225552 10.1016/s0167-8140(98)00147-9

[bib4] Bild AH, Potti A, Nevins JR (2006) Linking oncogenic pathways with therapeutic opportunities. Nat Rev Cancer 6: 735–741 16915294 10.1038/nrc1976

[bib5] Bourhis J, Overgaard J, Audry H, Ang KK, Saunders M, Bernier J, Horiot JC, Le Maitre A, Pajak TF, Poulsen MG, O'Sullivan B, Dobrowsky W, Hliniak A, Skladowski K, Hay JH, Pinto LH, Fallai C, Fu KK, Sylvester R, Pignon JP (2006) Hyperfractionated or accelerated radiotherapy in head and neck cancer: a meta-analysis. Lancet 368: 843–854 16950362 10.1016/S0140-6736(06)69121-6

[bib6] Caly M, Genin P, Ghuzlan AA, Elie C, Freneaux P, Klijanienko J, Rosty C, Sigal-Zafrani B, Vincent-Salomon A, Douggaz A, Zidane M, Sastre-Garau X (2004) Analysis of correlation between mitotic index, MIB1 score and S-phase fraction as proliferation markers in invasive breast carcinoma. Methodological aspects and prognostic value in a series of 257 cases. Anticancer Res 24: 3283–3288 15510624

[bib7] Chang HY, Sneddon JB, Alizadeh AA, Sood R, West RB, Montgomery K, Chi JT, van de Rijn M, Botstein D, Brown PO (2004) Gene expression signature of fibroblast serum response predicts human cancer progression: similarities between tumors and wounds. PLoS Biol 2: E7 14737219 10.1371/journal.pbio.0020007 PMC314300

[bib8] Chen HY, Yu SL, Chen CH, Chang GC, Chen CY, Yuan A, Cheng CL, Wang CH, Terng HJ, Kao SF, Chan WK, Li HN, Liu CC, Singh S, Chen WJ, Chen JJ, Yang PC (2007) A five-gene signature and clinical outcome in non-small-cell lung cancer. N Engl J Med 356: 11–20 17202451 10.1056/NEJMoa060096

[bib9] Chi JT, Wang Z, Nuyten DS, Rodriguez EH, Schaner ME, Salim A, Wang Y, Kristensen GB, Helland A, Borresen-Dale AL, Giaccia A, Longaker MT, Hastie T, Yang GP, van de Vijver MJ, Brown PO (2006) Gene expression programs in response to hypoxia: cell type specificity and prognostic significance in human cancers. PLoS Med 3: e47 16417408 10.1371/journal.pmed.0030047 PMC1334226

[bib10] Corvo R, Paoli G, Giaretti W, Sanguineti G, Geido E, Benasso M, Margarino G, Vitale V (2000) Evidence of cell kinetics as predictive factor of response to radiotherapy alone or chemoradiotherapy in patients with advanced head and neck cancer. Int J Radiat Oncol Biol Phys 47: 57–63 10758305 10.1016/s0360-3016(00)00416-8

[bib11] Dai H, van't Veer L, Lamb J, He YD, Mao M, Fine BM, Bernards R, van de Vijver M, Deutsch P, Sachs A, Stoughton R, Friend S (2005) A cell proliferation signature is a marker of extremely poor outcome in a subpopulation of breast cancer patients. Cancer Res 65: 4059–4066 15899795 10.1158/0008-5472.CAN-04-3953

[bib12] Daniels JM, Eerenberg JP, Rijna H, Kummer JA, Broeckaert MA, Paul MA, van Diest PJ, van Mourik JC (2002) Mitotic index does not predict prognosis in stage IA non-small cell lung cancer. Lung Cancer 38: 163–167 12399128 10.1016/s0169-5002(02)00215-5

[bib13] De Ruysscher D, Pijls-Johannesma M, Bentzen SM, Minken A, Wanders R, Lutgens L, Hochstenbag M, Boersma L, Wouters B, Lammering G, Vansteenkiste J, Lambin P (2006) Time between the first day of chemotherapy and the last day of chest radiation is the most important predictor of survival in limited-disease small-cell lung cancer. J Clin Oncol 24: 1057–1063 16505424 10.1200/JCO.2005.02.9793

[bib14] Dupuy A, Simon RM (2007) Critical review of published microarray studies for cancer outcome and guidelines on statistical analysis and reporting. J Natl Cancer Inst 99: 147–157 17227998 10.1093/jnci/djk018

[bib15] Dyrskjot L, Zieger K, Kruhoffer M, Thykjaer T, Jensen JL, Primdahl H, Aziz N, Marcussen N, Moller K, Orntoft TF (2005) A molecular signature in superficial bladder carcinoma predicts clinical outcome. Clin Cancer Res 11: 4029–4036 15930337 10.1158/1078-0432.CCR-04-2095

[bib16] Fan C, Oh DS, Wessels L, Weigelt B, Nuyten DS, Nobel AB, van′t Veer LJ, Perou CM (2006) Concordance among gene-expression-based predictors for breast cancer. N Engl J Med 355: 560–569 16899776 10.1056/NEJMoa052933

[bib17] Gasinska A, Fowler JF, Lind BK, Urbanski K (2004) Influence of overall treatment time and radiobiological parameters on biologically effective doses in cervical cancer patients treated with radiation therapy alone. Acta Oncol 43: 657–666 15545186 10.1080/02841860410018511

[bib18] Jalava P, Kuopio T, Juntti-Patinen L, Kotkansalo T, Kronqvist P, Collan Y (2006) Ki67 immunohistochemistry: a valuable marker in prognostication but with a risk of misclassification: proliferation subgroups formed based on Ki67 immunoreactivity and standardized mitotic index. Histopathology 48: 674–682 16681683 10.1111/j.1365-2559.2006.02402.x

[bib19] Larsen JE, Pavey SJ, Passmore LH, Bowman RV, Hayward NK, Fong KM (2007) Gene expression signature predicts recurrence in lung adenocarcinoma. Clin Cancer Res 13: 2946–2954 17504995 10.1158/1078-0432.CCR-06-2525

[bib20] Liu R, Wang X, Chen GY, Dalerba P, Gurney A, Hoey T, Sherlock G, Lewicki J, Shedden K, Clarke MF (2007) The prognostic role of a gene signature from tumorigenic breast-cancer cells. N Engl J Med 356: 217–226 17229949 10.1056/NEJMoa063994

[bib21] Miller LD, Smeds J, George J, Vega VB, Vergara L, Ploner A, Pawitan Y, Hall P, Klaar S, Liu ET, Bergh J (2005) An expression signature for p53 status in human breast cancer predicts mutation status, transcriptional effects, and patient survival. Proc Natl Acad Sci USA 102: 13550–13555 16141321 10.1073/pnas.0506230102 PMC1197273

[bib22] Paik S, Shak S, Tang G, Kim C, Baker J, Cronin M, Baehner FL, Walker MG, Watson D, Park T, Hiller W, Fisher ER, Wickerham DL, Bryant J, Wolmark N (2004) A multigene assay to predict recurrence of tamoxifen-treated, node-negative breast cancer. N Engl J Med 351: 2817–2826 15591335 10.1056/NEJMoa041588

[bib23] Perou CM, Jeffrey SS, van de Rijn M, Rees CA, Eisen MB, Ross DT, Pergamenschikov A, Williams CF, Zhu SX, Lee JC, Lashkari D, Shalon D, Brown PO, Botstein D (1999) Distinctive gene expression patterns in human mammary epithelial cells and breast cancers. Proc Natl Acad Sci USA 96: 9212–9217 10430922 10.1073/pnas.96.16.9212 PMC17759

[bib24] Quackenbush J (2006) Microarray analysis and tumor classification. N Engl J Med 354: 2463–2472 16760446 10.1056/NEJMra042342

[bib25] Rosenwald A, Wright G, Wiestner A, Chan WC, Connors JM, Campo E, Gascoyne RD, Grogan TM, Muller-Hermelink HK, Smeland EB, Chiorazzi M, Giltnane JM, Hurt EM, Zhao H, Averett L, Henrickson S, Yang L, Powell J, Wilson WH, Jaffe ES, Simon R, Klausner RD, Montserrat E, Bosch F, Greiner TC, Weisenburger DD, Sanger WG, Dave BJ, Lynch JC, Vose J, Armitage JO, Fisher RI, Miller TP, LeBlanc M, Ott G, Kvaloy S, Holte H, Delabie J, Staudt LM (2003) The proliferation gene expression signature is a quantitative integrator of oncogenic events that predicts survival in mantle cell lymphoma. Cancer Cell 3: 185–197 12620412 10.1016/s1535-6108(03)00028-x

[bib26] Sotiriou C, Wirapati P, Loi S, Harris A, Fox S, Smeds J, Nordgren H, Farmer P, Praz V, Haibe-Kains B, Desmedt C, Larsimont D, Cardoso F, Peterse H, Nuyten D, Buyse M, Van de Vijver MJ, Bergh J, Piccart M, Delorenzi M (2006) Gene expression profiling in breast cancer: understanding the molecular basis of histologic grade to improve prognosis. J Natl Cancer Inst 98: 262–272 16478745 10.1093/jnci/djj052

[bib27] Sung FL, Hui EP, Tao Q, Li H, Tsui NB, Dennis Lo YM, Ma BB, To KF, Harris AL, Chan AT (2007) Genome-wide expression analysis using microarray identified complex signaling pathways modulated by hypoxia in nasopharyngeal carcinoma. Cancer Lett 253(1): 74–88 17320280 10.1016/j.canlet.2007.01.012

[bib28] van 't Veer LJ, Dai H, van de Vijver MJ, He YD, Hart AA, Mao M, Peterse HL, van der Kooy K, Marton MJ, Witteveen AT, Schreiber GJ, Kerkhoven RM, Roberts C, Linsley PS, Bernards R, Friend SH (2002) Gene expression profiling predicts clinical outcome of breast cancer. Nature 415: 530–536 11823860 10.1038/415530a

[bib29] van de Vijver MJ, He YD, van't Veer LJ, Dai H, Hart AA, Voskuil DW, Schreiber GJ, Peterse JL, Roberts C, Marton MJ, Parrish M, Atsma D, Witteveen A, Glas A, Delahaye L, van der Velde T, Bartelink H, Rodenhuis S, Rutgers ET, Friend SH, Bernards R (2002) A gene-expression signature as a predictor of survival in breast cancer. N Engl J Med 347: 1999–2009 12490681 10.1056/NEJMoa021967

[bib30] Wang Y, Klijn JG, Zhang Y, Sieuwerts AM, Look MP, Yang F, Talantov D, Timmermans M, Meijer-van Gelder ME, Yu J, Jatkoe T, Berns EM, Atkins D, Foekens JA (2005) Gene-expression profiles to predict distant metastasis of lymph-node-negative primary breast cancer. Lancet 365: 671–679 15721472 10.1016/S0140-6736(05)17947-1

[bib31] Whitfield ML, George LK, Grant GD, Perou CM (2006) Common markers of proliferation. Nat Rev Cancer 6: 99–106 16491069 10.1038/nrc1802

[bib32] Whitfield ML, Sherlock G, Saldanha AJ, Murray JI, Ball CA, Alexander KE, Matese JC, Perou CM, Hurt MM, Brown PO, Botstein D (2002) Identification of genes periodically expressed in the human cell cycle and their expression in tumors. Mol Biol Cell 13: 1977–2000 12058064 10.1091/mbc.02-02-0030. PMC117619

[bib33] Winter SC, Buffa FM, Silva P, Miller C, Valentine HR, Turley H, Shah KA, Cox GJ, Corbridge RJ, Homer JJ, Musgrove B, Slevin N, Sloan P, Price P, West CM, Harris AL (2007) Relation of a hypoxia metagene derived from head and neck cancer to prognosis of multiple cancers. Cancer Res 67: 3441–3449 17409455 10.1158/0008-5472.CAN-06-3322

