# Supplementary material for: Robust prognostic value of a knowledge-based proliferation signature across large patient microarray studies spanning different cancer types
Source: Br J Cancer. 2008 Nov 4;99(11):1884–90. doi: 10.1038/sj.bjc.6604746 (PMC2600688; doi:10.1038/sj.bjc.6604746)
Supplement: Supplementary Materials and Methods [file 6604746x1.doc]

**Supplementary materials and methods**

*Signature processing*

For all signatures the gene identifiers were translated into UnigeneIDs (Build199) with Source (http://smd.stanford.edu/) or Affymetrix data files (www.affymetrix.com: Affx annotation files). After this translation several genes in the proliferation signatures were represented by more than one cloneID. In case these cloneIDs represented the same proliferation status they were included in the signature. However when multiple cloneIDs representing one gene corresponded to different proliferation conditions these genes were discarded. This was approximately 3% of the genes in each signature.

*Data filtering and pre-processing*

Datasets downloaded from the SMD (Zhao *et al*, 2006) were filtered according to the parameters in the paper. CloneIDs were chosen as gene annotation and the data obtained was log-transformed. For the normalized affymetrix arrays (Miller *et al*, 2005; Wang *et al*, 2005) the genes were log-transformed. The Beer *et al* (Beer *et al*, 2002) dataset was already preprocessed therefore to perform log-transformation all expression values below 1.1 were set to 1.1, this was similar to the processing performed by Chen *et al* (Chen *et al*, 2007). In all other cases the data was kept in the downloaded format (van de Vijver *et al*, 2002), which was already log-transformed.

CloneIDs and affymetrix probeIDs were translated into UnigeneIDs (Build199).

*Weight assignment*

Genes with a peak expression in S, G2 and M phase clearly are proliferation genes. Higher expression of these genes will correlate with a larger proportion of cells in S, G2 and M phase, which indicates more proliferation. That does not apply to genes with a peak expression in G1, since a G1 cell may or may not commit to cell cycle. Therefore genes with a peak expression in G1, 34% and 25% of the genes in signature 1 and 2 respectively, were omitted from further analyses. Equal weights were assigned to the remaining genes in the two proliferation signatures.

For the wound signature weights of -1 and 1 were assigned to genes representing a quiescent and activated wound response respectively. For the IGS signature log ratios were provided in the supplementary data of the paper, a weight of 1 is assigned to genes with a positive log ratio and a weight of -1 to genes with a negative log ratio.

*AUC model calculation*

Matlab (Matlab 7.1, The Mathworks, Massachusetts, USA) was used to integrate all parameters in a model and evaluate the area under the curve (AUC) of the model with and without addition of the signature to the clinical parameters. All clinical parameters were transformed to numbers, to be able to incorporate them in Matlab, e.g. negative and positive ER-status were set to 0 and 1 respectively. These parameters were incorporated in a model with the classify function of Matlab, which used the diaglinear method. Part of the dataset was used as training set and the other part as a test set. Assignment of samples to test and training set was done at random and repeated 1,000 times.

*Contingency table analyses*

Contingency tables were used to compare patient classification of the proliferation signature to the patient classification of other gene signatures. For three datasets (Beer *et al*, 2002; Miller *et al*, 2005; van de Vijver *et al*, 2002) we were able to find the group classification of the gene signatures identified in these studies: the 32-gene p53 signature (Miller *et al*, 2005), the 70-gene signature (van de Vijver *et al*, 2002) and the 100 survival related genes (Beer *et al*, 2002). These and the wound response and IGS signature were evaluated.

Contingency tables were evaluated with the p-value calculated from Chi-square test and the Cramer’s V statistic. The Cramer’s V statistic (value can range from 0 to 1) measures the strength of association between the two variables analyzed in the contingency table, with 1 indicating perfect association and 0 indicating no association. Values between 0.36 and 0.49 indicate a substantial relation between the signatures and values >0.50 indicate a strong relation (Fan *et al*, 2006).

**Supplementary tables**

**Table S1** Percentages of genes in the different cell cycle phases in the two proliferation signatures (numbers are given between brackets)

| **Cell cycle phase** | **Signature 1** | **Signature 2** |
| --- | --- | --- |
| **G1­/S** | 18.0 (147) | 18.8 (29) |
| **S** | 18.0 (147) | 19.5 (30) |
| **G2** | 19.5 (159) | 26.0 (40) |
| **G2/M** | 21.7 (177) | 22.1 (34) |
| **M/G1** | 16.0 (130) | 6.5 (10) |
| **Matching combinations*** | 3.1 (25) | 3.9 (6) |
| **Non-matching combinations†** | 3.7 (30) | 3.3 (5) |
| * Different cloneIDs for 1 UnigeneID are found in different phases, but all phases represent the same proliferation status (i.e S, G2, G2/M)  † Different cloneIDs for 1 UnigeneID are found in different phases and the phases represent a different proliferation status (i.e G2/M, G1/S) | | |

**Table S2** Results of log-rank test for the signatures for the different end-points

| **Signature 1** |  |  |  |  |  |
| --- | --- | --- | --- | --- | --- |
| **Dataset** | **End-point** | **P-value** | **SD** | **Range** | **% of significant runs** |
| **Miller** | 5-years | 3.1 10-3 | 1.6 10-3 | 1.5 10-3 – 4.7 10-3 | 100 |
|  | 10-years | 6.9 10-4 | 3.4 10-4 | 3.6 10-4 – 1.0 10-3 | 100 |
| **Wang** | 5-years | 1.9 10-3 | 1.0 10-3 | 9.9 10-4 – 3.0 10-3 | 100 |
| **Van de Vijver** | 5-years | 4.6 10-5 | 0.0 | 4.6 10-5 – 4.6 10-5 | 100 |
|  | 10-years | 2.5 10-7 | 0.0 | 2.5 10-7 – 2.5 10-7 | 100 |
| **Zhao** | 5-years | 0.48 | 8.0 10-2 | 0.39 – 0.55 | 0 |
|  | 10-years | 0.54 | 3.0 10-2 | 0.51 – 0.57 | 0 |
| **Beer** | 2-years | 0.16 | 5.4 10-2 | 0.12 – 0.20 | 0 |
|  | 5-years | 0.49 | 9.8 10-2 | 0.11 – 0.63 | 0 |
|  |  |  |  |  |  |
| **Signature 2** |  |  |  |  |  |
| **Dataset** | **End-point** | **P-value** | **SD** | **Range** | **% of significant runs** |
| **Miller** | 5-years | 4.1 10-3 | 1.8 10-3 | 2.0 10-3 – 6.2 10-3 | 100 |
|  | 10-years | 7.0 10-4 | 1.8 10-4 | 4.6 10-4 – 9.3 10-4 | 100 |
| **Wang** | 5-years | 2.3 10-3 | 1.8 10-4 | 5.7 10-5 – 6.4 10-4 | 100 |
| **Van de Vijver** | 5-years | 1.4 10-6 | 0.0 | 1.4 10-6 – 1.4 10-6 | 100 |
|  | 10-years | 3.0 10-8 | 1.6 10-10 | 3.0 10-8 – 3.0 10-8 | 100 |
| **Zhao** | 5-years | 3.1 10-2 | 1.1 10-2 | 1.9 10-2 – 4.2 10-2 | 100 |
|  | 10-years | 2.3 10-2 | 3.2 10-3 | 2.0 10-2 – 2.7 10-2 | 100 |
| **Beer** | 2-years | 3.3 10-3 | 2.2 10-5 | 3.3 10-3 – 3.4 10-3 | 100 |
|  | 5-years | 2.8 10-2 | 6.8 10-5 | 2.8 10-2 – 2.8 10-2 | 100 |
|  |  |  |  |  |  |
| **Wound signature** | |  |  |  |  |
| **Dataset** | **End-point** | **P-value** | **SD** | **Range** | **% of significant runs** |
| **Miller** | 5-years | 1.8 10-4 | 1.1 10-4 | 4.8 10-5 – 8.7 10-4 | 100 |
|  | 10-years | 9.3 10-6 | 3.9 10-6 | 3.1 10-6 – 3.9 10-5 | 100 |
| **Wang** | 5-years | 2.1 10-3 | 1.4 10-3 | 5.1 10-4 – 4.1 10-3 | 100 |
| **Van de Vijver** | 5-years | 5.9 10-5 | 0.0 | 5.9 10-5 – 5.9 10-5 | 100 |
|  | 10-years | 9.3 10-7 | 5.2 10-9 | 9.3 10-7 – 9.4 10-7 | 100 |
| **Zhao** | 5-years | 2.1 10-2 | 6.9 10-3 | 2.5 10-2 – 3.1 10-2 | 100 |
|  | 10-years | 2.8 10-2 | 2.7 10-3 | 1.4 10-2 – 2.8 10-2 | 100 |
| **Beer** | 2-years | 6.9 10-2 | 5.1 10-2 | 1.5 10-2 – 0.10 | 49 |
|  | 5-years | 0.12 | 1.2 10-2 | 8.1 10-2 – 0.13 | 0 |
|  |  |  |  |  |  |
| **IGS signature** |  |  |  |  |  |
| **Dataset** | **End-point** | **P-value** | **SD** | **Range** | **% of significant runs** |
| **Miller** | 5-years | 5.3 10-3 | 2.6 10-3 | 2.8 10-3 – 8.1 10-3 | 100 |
|  | 10-years | 2.4 10-3 | 7.4 10-4 | 1.7 10-3 – 3.2 10-3 | 100 |
| **Wang** | 5-years | 2.1 10-6 | 1.7 10-6 | 4.3 10-7 – 5.1 10-6 | 100 |
| **Van de Vijver** | 5-years | 1.5 10-4 | 0.0 | 1.5 10-4 – 1.5 10-4 | 100 |
|  | 10-years | 4.9 10-7 | 0.0 | 4.9 10-7 – 4.9 10-7 | 100 |
| **Zhao** | 5-years | 2.2 10-3 | 1.0 10-3 | 1.0 10-3 – 3.4 10-3 | 100 |
|  | 10-years | 4.7 10-3 | 9.5 10-4 | 3.3 10-3 – 5.9 10-3 | 100 |
| **Beer** | 2-years | 2.8 10-2 | 6.6 10-3 | 2.3 10-2 – 3.7 10-2 | 100 |
|  | 5-years | 5.4 10-2 | 5.4 10-3 | 5.0 10-2 – 6.1 10-2 | 61 |
|  |  |  |  |  |  |

**Table S3** AUCs of individual clinical parameters and proliferation signature 2

| **Miller** | | **Wang** | | **Van de Vijver** | | **Zhao** | | **Beer** | |
| --- | --- | --- | --- | --- | --- | --- | --- | --- | --- |
| Age  Elston grade  Tumor size*  ER-status  LNS‡  PgR§  P53-status  Proliferation** | 0.48  0.65  0.71  0.58  0.70  0.54  0.57  0.67 | ER-status  Proliferation** | 0.62  0.59 | Age  Elston grade  Tumor size*  ER-status  LNS‡  NIH risk  Mastectomy  Chemotherapy  Hormone therapy  Proliferation** | 0.42  0.72  0.67  0.41  0.52  0.66  0.57  0.52  0.53  0.72 | Age  Sex  Performance status  Grade  Stage  Proliferation** | 0.43  0.45  0.60  0.64  0.85  0.56 | Age  Sex  Smoking**†**  Stage  Differentiation  K-ras mutation  Proliferation** | 0.60  0.45  0.49  0.66  0.59  0.52  0.64 |
| * Categories: ≤ 2 cm or > 2 cm  **†** Categories: smoker or non-smoker  ‡ LNS: lymph-node status  § PgR: progesterone receptor status  ** proliferation: proliferation signature 2 | | | | | | | | | |

**Table S4** Parameters selected with stepwise backward selection in multivariate Cox-regression analyses including signature 2, wound and IGS signature

|  |  | **Input** |  |  |  |  |
| --- | --- | --- | --- | --- | --- | --- |
| **Dataset** | **Endpoint** | **Clinical parameters** | **Clinical parameters +**  **Proliferation**  **Signature 2** | **Clinical parameters +**  **Proliferation signature 2 +**  **Wound signature** | **Clinical parameters +**  **Proliferation signature 2 +**  **IGS** | **Signatures** |
| **Miller** | **5 years** | Tumor size*  LNS^ | Tumor size*  LNS^  Age  Proliferation+ | Tumor size*  LNS^  Age  Wound | Tumor size*  LNS^  Age  Proliferation+ | Wound |
|  | **10 years** | Tumor size*  LNS^  p53-status | Tumor size*  LNS^  Proliferation+ | Tumor size*  LNS^  Age  Wound | Tumor size*  LNS^  Proliferation+  IGS | Wound |
| **Wang** | **5 years** | ER-status | Proliferation+ | Proliferation+ | IGS | IGS |
| **van de Vijver** | **5 years** | Tumor size*  ER-status  Elston grade | Tumor size*  ER-status  Elston grade | Tumor size*  ER-status  Elston grade | Tumor size*  ER-status  Elston grade | Proliferation+  IGS |
|  | **10 years** | Tumor size*  ER-status  Elston grade  Age | Tumor size*  Elston grade  Age  Proliferation+ | Tumor size*  Elston grade  Age  Proliferation+ | Tumor size*  ER-status  Elston grade  Age  IGS | Proliferation+  IGS |
| **Zhao** | **5 years** | Stage  Grade  Performance status | Stage  Grade  Performance status | Stage  Grade  Performance status | Stage  Grade  Performance status  Proliferation+  IGS | Proliferation+  Wound  IGS |
|  | **10 years** | Stage  Grade  Performance status | Stage  Grade  Performance status | Stage  Grade  Performance status | Stage  Grade  Performance status | Proliferation+  Wound  IGS |
| **Beer** | **2 years** | Stage  Differentiation | Stage  Differentiation | Stage  Differentiation | Stage  Differentiation | Proliferation+ |
|  | **5 years** | Stage  Age  Differentiation | Stage  Age  Proliferation+ | Stage  Age  Proliferation+ | Stage  Age  Proliferation+ | Proliferation+ |
| * Categories: ≤ 2 cm or > 2 cm  ^ LNS: lymph-node status  + proliferation: proliferation signature 2 | | | | | | |

**Table S5** Two-way contingency table analyses measuring the association between the proliferation signature and the wound (A), IGS (B) and 32-gene p53 signature (C) in the Miller dataset

| **A** | | **Wound signature** | | |
| --- | --- | --- | --- | --- |
|  |  | **Quiescent** | **Activated** | |
| **Proliferation signature** | **Low score** | 120 | 15 | |
|  | **High score** | 19 | 82 | |
| Statistics:  p-value >0.001  Cramer’s V 0.70 |  |  |  | |
|  | | | | |
| **B** | | **IGS signature** | | |
|  |  | **Low score** | **High score** | |
| **Proliferation signature** | **Low score** | 105 | 30 | |
|  | **High score** | 19 | 82 | |
| Statistics:  p-value >0.001  Cramer’s V 0.58 |  |  |  | |
|  | | | | |
| **C** | | **32-gene p53 signature** | | |
|  |  | **p53-wt like** | | **P53-mu like** |
| **Proliferation signature** | **Low score** | 125 | | 43 |
|  | **High score** | 10 | | 58 |
| Statistics:  p-value >0.001  Cramer’s V 0.55 |  |  | |  |

**Table S6** Two-way contingency table analyses measuring the association between the proliferation signature and the wound (A) and IGS (B) signature in the Wang dataset

| **A** | | **Wound signature** | | |
| --- | --- | --- | --- | --- |
|  |  | **Quiescent** | **Activated** | |
| **Proliferation signature** | **Low score** | 124 | 12 | |
|  | **High score** | 60 | 90 | |
| Statistics:  p-value >0.001  Cramer’s V 0.53 |  |  |  | |
|  | | | | |
| **B** | | **IGS signature** | | |
|  |  | **Low score** | | **High score** |
| **Proliferation signature** | **Low score** | 91 | | 45 |
|  | **High score** | 27 | | 123 |
| Statistics:  p-value >0.001  Cramer’s V 0.50 |  |  | |  |

**Table S7** Two-way contingency table analyses measuring the association between the proliferation signature and the wound (A), IGS (B) and 70-gene (C) signature in the van de Vijver dataset

| **A** | | **Wound signature** | |
| --- | --- | --- | --- |
|  |  | **Quiescent** | **Activated** |
| **Proliferation signature** | **Low score** | 150 | 10 |
|  | **High score** | 25 | 110 |
| Statistics:  p-value >0.001  Cramer’s V 0.76 |  |  |  |
|  | | | |
| **B** | | **IGS signature** | |
|  |  | **Low score** | **High score** |
| **Proliferation signature** | **Low score** | 114 | 46 |
|  | **High score** | 34 | 101 |
| Statistics:  p-value >0.001  Cramer’s V 0.46 |  |  |  |
|  | | | |
| **C** | | **70-gene signature** | |
|  |  | **Poor profile** | **Good profile** |
| **Proliferation signature** | **Low score** | 106 | 54 |
|  | **High score** | 9 | 126 |
| Statistics:  p-value >0.001  Cramer’s V 0.61 |  |  |  |

**Table S8** Two-way contingency table analyses measuring the association between the proliferation signature and the wound (A) and IGS (B) signature in the Zhao dataset

| **A** | | **Wound signature** | |
| --- | --- | --- | --- |
|  |  | **Quiescent** | **Activated** |
| **Proliferation signature** | **Low score** | 84 | 11 |
|  | **High score** | 49 | 33 |
| Statistics:  p-value >0.001  Cramer’s V 0.33 |  |  |  |
|  | | | |
| **B** | | **IGS signature** | |
|  |  | **Low score** | **High score** |
| **Proliferation signature** | **Low score** | 77 | 18 |
|  | **High score** | 30 | 52 |
| Statistics:  p-value >0.001  Cramer’s V 0.45 |  |  |  |

**Table S9** Two-way contingency table analyses measuring the association between the proliferation signature and the wound (A), IGS (B) and 100 survival related genes (C) in the Beer dataset

| **A** | | **Wound signature** | |
| --- | --- | --- | --- |
|  |  | **Quiescent** | **Activated** |
| **Proliferation signature** | **Low score** | 42 | 3 |
|  | **High score** | 6 | 35 |
| Statistics:  p-value >0.001  Cramer’s V 0.78 |  |  |  |
|  | | | |
| **B** | | **IGS signature** | |
|  |  | **Low score** | **High score** |
| **Proliferation signature** | **Low score** | 39 | 6 |
|  | **High score** | 14 | 27 |
| Statistics:  p-value >0.001  Cramer’s V 0.54 |  |  |  |
|  | | | |
| **C** | | **100 survival related genes** | |
|  |  | **Low risk** | **High risk** |
| **Proliferation signature** | **Low score** | 33 | 12 |
|  | **High score** | 10 | 31 |
| Statistics:  p-value >0.001  Cramer’s V 0.50 |  |  |  |

**References**

Beer DG, Kardia SL, Huang CC, Giordano TJ, Levin AM, Misek DE, Lin L, Chen G, Gharib TG, Thomas DG, Lizyness ML, Kuick R, Hayasaka S, Taylor JM, Iannettoni MD, Orringer MB, Hanash S (2002) Gene-expression profiles predict survival of patients with lung adenocarcinoma. *Nat Med*, **8:** 816-24.

Chen HY, Yu SL, Chen CH, Chang GC, Chen CY, Yuan A, Cheng CL, Wang CH, Terng HJ, Kao SF, Chan WK, Li HN, Liu CC, Singh S, Chen WJ, Chen JJ, Yang PC (2007) A five-gene signature and clinical outcome in non-small-cell lung cancer. *N Engl J Med*, **356:** 11-20.

Fan C, Oh DS, Wessels L, Weigelt B, Nuyten DS, Nobel AB, van't Veer LJ, Perou CM (2006) Concordance among gene-expression-based predictors for breast cancer. *N Engl J Med*, **355:** 560-9.

Miller LD, Smeds J, George J, Vega VB, Vergara L, Ploner A, Pawitan Y, Hall P, Klaar S, Liu ET, Bergh J (2005) An expression signature for p53 status in human breast cancer predicts mutation status, transcriptional effects, and patient survival. *Proc Natl Acad Sci U S A*, **102:** 13550-5.

van de Vijver MJ, He YD, van't Veer LJ, Dai H, Hart AA, Voskuil DW, Schreiber GJ, Peterse JL, Roberts C, Marton MJ, Parrish M, Atsma D, Witteveen A, Glas A, Delahaye L, van der Velde T, Bartelink H, Rodenhuis S, Rutgers ET, Friend SH, Bernards R (2002) A gene-expression signature as a predictor of survival in breast cancer. *N Engl J Med*, **347:** 1999-2009.

Wang Y, Klijn JG, Zhang Y, Sieuwerts AM, Look MP, Yang F, Talantov D, Timmermans M, Meijer-van Gelder ME, Yu J, Jatkoe T, Berns EM, Atkins D, Foekens JA (2005) Gene-expression profiles to predict distant metastasis of lymph-node-negative primary breast cancer. *Lancet*, **365:** 671-9.

Zhao H, Ljungberg B, Grankvist K, Rasmuson T, Tibshirani R, Brooks JD (2006) Gene expression profiling predicts survival in conventional renal cell carcinoma. *PLoS Med*, **3:** e13.
